# Mendelian randomization integrating GWAS and eQTL data revealed genes pleiotropically associated with major depressive disorder

**DOI:** 10.1038/s41398-021-01348-0

**Published:** 2021-04-17

**Authors:** Huarong Yang, Di Liu, Chuntao Zhao, Bowen Feng, Wenjin Lu, Xiaohan Yang, Minglu Xu, Weizhu Zhou, Huiquan Jing, Jingyun Yang

**Affiliations:** 1 Department of Neurology, The Second Affiliated Hospital of Guizhou Medical University, Kaili, Guizhou China; 2 Beijing Key Laboratory of Clinical Epidemiology, School of Public Health, Capital Medical University, Beijing, China; 3 Brain Tumor Center, Cancer & Blood Diseases Institute, Cincinnati Children’s Hospital Medical Center, Cincinnati, OH USA; 4 Odette School of Business, University of Windsor, Windsor, ON Canada; 5 Department of Mathematics, University College London, London, UK; 6 Department of Health Management and Policy, School of Public Health, Capital Medical University, Beijing, China; 7 Department of Epidemiology and Health Statistics, School of Public Health, Shenyang Medical College, Shenyang, China; 8 Rush Alzheimer’s Disease Center, Rush University Medical Center, Chicago, IL USA; 9 Department of Neurological Sciences, Rush University Medical Center, Chicago, IL USA

**Keywords:** Molecular neuroscience, Comparative genomics

## Abstract

Previous genome-wide association studies (GWAS) have identified potential genetic variants associated with the risk of major depressive disorder (MDD), but the underlying biological interpretation remains largely unknown. We aimed to prioritize genes that were pleiotropically or potentially causally associated with MDD. We applied the summary data-based Mendelian randomization (SMR) method integrating GWAS and gene expression quantitative trait loci (eQTL) data in 13 brain regions to identify genes that were pleiotropically associated with MDD. In addition, we repeated the analysis by using the meta-analyzed version of the eQTL summary data in the brain (brain-eMeta). We identified multiple significant genes across different brain regions that may be involved in the pathogenesis of MDD. The prime-specific gene *BTN3A2* (corresponding probe: ENSG00000186470.9) was the top hit showing pleiotropic association with MDD in 9 of the 13 brain regions and in brain-eMeta, after correction for multiple testing. Many of the identified genes are located in the human major histocompatibility complex (MHC) region on chromosome 6 and are mainly involved in the immune response. Our SMR analysis indicated that multiple genes showed pleiotropic association with MDD across the brain regions. These findings provided important leads to a better understanding of the mechanism of MDD and revealed potential therapeutic targets for the prevention and effective treatment of MDD.

## Introduction

Major depressive disorder (MDD) is a significant medical condition impacting an individual’s mood, behavior, appetite, and sleep as well as thoughts of suicide^1^. MDD is a leading cause of disability and morbidity worldwide^2^, with an estimated lifetime prevalence of around 15%^3^. MDD is a complex multifactorial disorder, with contributions from both genetic and environmental factors^4^. However, the exact etiology of MDD remains to be unclear, and there is pressing urgency to further explore the pathological mechanisms underlying MDD to facilitate the design and implementation of efficient prevention strategies or novel treatments.

Previous twin studies found the heritability of MDD to be ~30–40%^5,6^. Genome-wide association studies (GWASs) have been successful in identifying genetic variants associated with MDD^7–11^, such as single-nucleotide polymorphisms (SNPs) in/near sortilin-related VPS10-domain-containing receptor 3 (*SORCS3*), transcription factor 4 (*TCF4*), and neuronal growth regulator 1 (*NEGR1*). However, the biological interpretation of the identified genetic variants remains largely unclear. Because many of the genetic variants identified in GWASs are located in non-coding regions, it is likely that these genetic variants exert their effects on diseases/disorders via gene expression^12^. Therefore, it is important to explore the relationship between genetic variation and gene expression to better understand the regulatory pathways underlying the pathogenesis of MDD.

Mendelian randomization (MR) is a method for exploring the potential causal association between an exposure and an outcome by using genetic variants as the instrumental variables (IVs) for exposure^13^. Compared with traditional statistical methods used in the association studies, MR reduces confounding and reverse causation and is becoming increasingly popular in the exploration of etiological mechanisms^14,15^. A novel analytical framework through a summary data-based MR (SMR) approach integrating *cis*-expression quantitative trait loci (*cis*-eQTL) or *cis*-DNA methylation QTL (*cis*-mQTL) and GWAS data have been successful in identifying gene expressions or DNA methylation loci that are pleiotropically or potentially causally associated with various phenotypes, such as cardiovascular diseases, systemic lupus erythematosus, inflammatory bowel disease, and educational attainment^16–20^, indicating that it is a promising tool to explore genes pleiotropically associated with complex traits.

Previous research that adopted the SMR approach to examine possible causal genes for MDD found three candidate genes (*NEGR1*, *BAG6*, and *HLG-B*). The GWAS data used in the study were based on 42,455 subjects (16,823 MDD cases and 25,632 controls), and the *cis*-eQTL data were based on a meta-analyzed version of the eQTL summary data instead of specific brain regions^21^. In this study, we adopted the SMR approach by leveraging GWAS summarized results for MDD which were based on a much larger sample size (807,553 subjects), and *cis*-eQTL data in 13 different brain regions to prioritize genes that are pleiotropically or potentially causally associated with MDD across different brain regions.

## Materials and methods

In the SMR analysis, *cis*-eQTL genetic variants were used as the IVs for gene expression. We performed SMR analysis for different regions in the brain. We used the Version 7 release of the eQTL summarized data from the Genotype Tissue Expression (GTEx)^22^ project, which included 13 different regions: amygdala, anterior cingulate cortex, caudate nucleus, cerebellar hemisphere, cerebellum, cortex, frontal cortex, hippocampus, hypothalamus, nucleus accumbens, putamen, spinal cord, and substantia nigra^22^. In addition, we repeated the analysis by using the meta-analyzed version of the eQTL summary data (named brain-eMeta hereafter), which included results from the GTEx data of brain tissues^22^, the Common Mind Consortium^23^, and the Religious Orders Study and the Rush Memory and Aging Project^24^. Results from these three studies were meta-analyzed using the MeCS method (meta-analysis of *cis*-eQTL in correlated samples) to increase the power of detecting brain eQTLs^25^. Only SNPs within 1 Mb distance from each individual probe are available. The eQTL data can be downloaded at https://cnsgenomics.com/data/SMR/#eQTLsummarydata.

The GWAS summarized data for MDD were provided by the Psychiatric Genomics Consortium^10^. The results were based on three large genome-wide association studies^8,9,11^, including a total of 807,553 individuals (246,363 cases and 561,190 controls, after excluding overlapping samples) and 8,098,588 genetic variants. The GWAS summarized data can be downloaded at https://www.med.unc.edu/pgc/download-results/mdd/.

MR was carried out considering *cis*-eQTL genetic variants as the IVs, gene expression as the exposure, and MDD as the outcome. MR analysis was performed using the method as implemented in the software SMR. Detailed information regarding the SMR method has been described previously^16^. Briefly, SMR uses the principles of MR integrating GWAS and eQTL summary statistics to test for pleiotropic association between gene expression and MDD due to a shared and potentially causal variant at a locus. The heterogeneity in dependent instruments (HEIDI) test was done to explore the existence of linkage in the observed association. Rejection of the null hypothesis (i.e., *P*_HEIDI_ < 0.05) indicates that the observed association might be due to two distinct genetic variants in high linkage disequilibrium with each other. We adopted the default settings in SMR (e.g., *P*_eQTL_ < 5 × 10^−^^8^, minor allele frequency [MAF] > 0.01, excluding SNPs in very strong linkage disequilibrium [LD, *r*^2^ > 0.9] with the top associated eQTL, and removing SNPs in low LD or not in LD [*r*^2^ < 0.05] with the top associated eQTL), and used false discovery rate (FDR) to adjust for multiple testing.

Annotations of the transcripts were based on the Affymetrix exon array S1.0 platforms. To functionally annotate putative transcripts, we conducted functional enrichment analysis using the functional annotation tool “Metascape” for the significant genes in different brain regions and in brain-eMeta. Gene symbols corresponding to putative genes (FDR *P* < 0.05) were used as the input of the gene ontology (GO) and Kyoto Encyclopedia of Genes and Genomes (KEGG) enrichment analysis.

Data cleaning and statistical/bioinformatical analysis were performed using R version 4.0.2 (https://www.r-project.org/), PLINK 1.9 (https://www.cog-genomics.org/plink/1.9/) and SMR (https://cnsgenomics.com/software/smr/).

## Results

The number of participants used for generating the eQTL data varied across the brain regions, ranging from 114 to 209, so did the number of eligible probes involved in the final SMR analysis, ranging from 661 to 3765. The brain-eMeta analysis involved more subjects (*n* = 1194) and more probes (*n* = 7421). The GWAS meta-analysis data involved roughly 800,000 subjects. The detailed information was shown in Table 1.

**Table 1:** Basic information of the eQTL and GWAS data.

Data source	Total participants or cases/controls	Number of genetic variants or probes
eQTL data		
Amygdala	129	779
Anterior cingulate cortex	147	1379
Caudate	194	2089
Cerebellar hemisphere	175	2615
Cerebellum	209	3765
Cortex	205	2314
Frontal cortex	175	1722
Hippocampus	165	1108
Hypothalamus	170	1170
Nucleus accumbens	202	1785
Putamen	170	1449
Spinal cord	126	915
Substantia nigra	114	661
Brain-eMeta	1194	7421
GWAS-Meta	246,363/561,190	8,098,588
23andme_307k	75,607/231,747	
UK Biobank	127,552/233,763	
PGC_139k	43,204/95,680	

*GWAS* genome-wide association studies, *QTL* quantitative trait loci, *PGC* Psychiatric Genomics Consortium.

We identified multiple genes showing pleiotropic association with MDD across the different brain regions (Table 2 and Supplementary Fig. S1). Out of the 13 brain regions, the human major histocompatibility complex (MHC) gene *BTN3A2* (ENSG00000186470.9) was the top hit showing pleiotropic association with MDD in 9 regions, after correction for multiple testing. Each of the other two genes, *RPL31P12* (ENSG00000227207.2) and *RP1-265C24.5* (ENSG00000219392.1) was the top gene pleiotropically associated with MDD in two brain regions (Table 2).

**Table 2:** Summary of the SMR analyses across the 13 brain regions.

Regions	Number of genes	Top probe	Tope gene	CHR	Top SNP	*P*_SMR_	*Q*_value
Amygdala	5	ENSG00000186470.9	*BTN3A2*	6	rs9393703	3.08 × 10^−7^	2.40 × 10^−^^4^
Anterior cingulate cortex	5	ENSG00000186470.9	*BTN3A2*	6	rs28551159	2.46 × 10^−^^7^	3.40 × 10^−^^4^
Caudate	7	ENSG00000186470.9	*BTN3A2*	6	rs9379853	7.76 × 10^−^^8^	1.62 × 10^−^^4^
Cerebellar hemisphere	21	ENSG00000227207.2	*RPL31P12*	1	rs1460943	7.53 × 10^−^^11^	1.97 × 10^−^^7^
Cerebellum	30	ENSG00000227207.2	*RPL31P12*	1	rs1460943	1.34 × 10^−^^12^	5.03 × 10^−^^9^
Cortex	7	ENSG00000219392.1	*RP1-265C24.5*	6	rs2295594	6.13 × 10^−^^8^	1.42 × 10^−^^4^
Frontal cortex	4	ENSG00000186470.9	*BTN3A2*	6	rs9379853	2.20 × 10^−^^7^	3.78 × 10^−^^4^
Hippocampus	3	ENSG00000186470.9	*BTN3A2*	6	rs9393703	5.32 × 10^−^^7^	5.90 × 10^−^^4^
Hypothalamus	3	ENSG00000186470.9	*BTN3A2*	6	rs72841536	5.34 × 10^−^^7^	6.25 × 10^−^^4^
Nucleus accumbens	3	ENSG00000219392.1	*RP1-265C24.5*	6	rs4713135	1.63 × 10^−^^8^	2.91 × 10^−^^5^
Putamen	2	ENSG00000186470.9	*BTN3A2*	6	rs9379853	3.41 × 10^−^^7^	4.95 × 10^−^^4^
Spinal cord	2	ENSG00000186470.9	*BTN3A2*	6	rs71557332	1.72 × 10^−^^7^	1.58 × 10^−^^4^
Substantia nigra	1	ENSG00000186470.9	*BTN3A2*	6	rs28551159	4.42 × 10^−^^6^	2.92 × 10^−^^3^

Number of genes means the number of statistically significant genes in each region after correction for multiple testing using false discovery rate (*Q* value < 0.05); top probe and gene is the probe and the corresponding gene having the smallest *P*_SMR_ in the region; top SNP is the top associated *cis*-eQTL for the corresponding probe in the eQTL analysis; *P*_SMR_ is the *P-*value for SMR analysis.

*CHR* chromosome, *SNP* single-nucleotide polymorphism, *SMR* summary data-based Mendelian randomization, *QTL* quantitative trait loci.

**Fig. 1:**
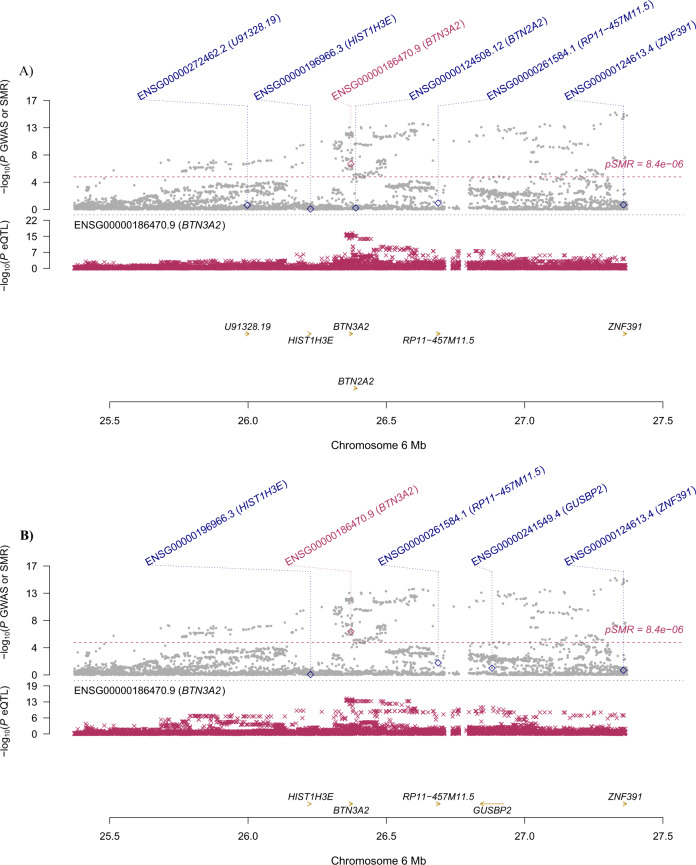
Prioritizing genes around *BTN3A2* in pleiotropic association with MDD. **A** Caudate nucleus. **B** Spinal cord. Top plot, gray dots represent the −log_10_(*P* values) for SNPs from the GWAS of MDD, and rhombuses represent the −log_10_(*P* values) for probes from the SMR test with solid rhombuses indicating that the probes pass HEIDI test and hollow rhombuses indicating that the probes do not pass the HEIDI test. Middle plot, eQTL results for the probe ENSG000001864770.9 tagging *BTN3A2*. Bottom plot, location of genes tagged by the probe. Highlighted in maroon indicates probes that pass the SMR threshold. GWAS genome-wide association study, MDD major depressive disorder, SMR summary data-based Mendelian randomization, HEIDI heterogeneity in dependent instruments, eQTL expression quantitative trait loci.

**Fig. 2:**
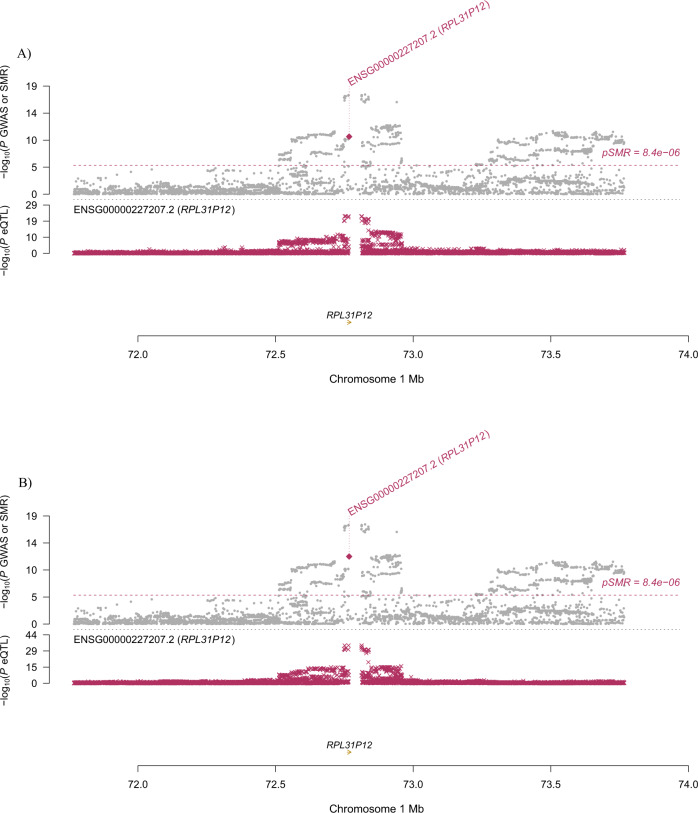
Prioritizing genes around *RPL31P12* in pleiotropic association with MDD. **A** Cerebellar hemisphere. **B** Cerebellum. Top plot, gray dots represent the −log_10_(*P* values) for SNPs from the GWAS of MDD, and rhombuses represent the −log_10_(*P* values) for probes from the SMR test with solid rhombuses indicating that the probes pass HEIDI test and hollow rhombuses indicating that the probes do not pass the HEIDI test. Middle plot, eQTL results for the probe ENSG00000227207.2 tagging *RPL31P12*. Bottom plot, location of genes tagged by the probe. Highlighted in maroon indicates probes that pass the SMR threshold. GWAS genome-wide association studies, MDD major depressive disorder, SMR summary data-based Mendelian randomization, HEIDI heterogeneity in dependent instruments, eQTL expression quantitative trait loci.

**Fig. 3:**
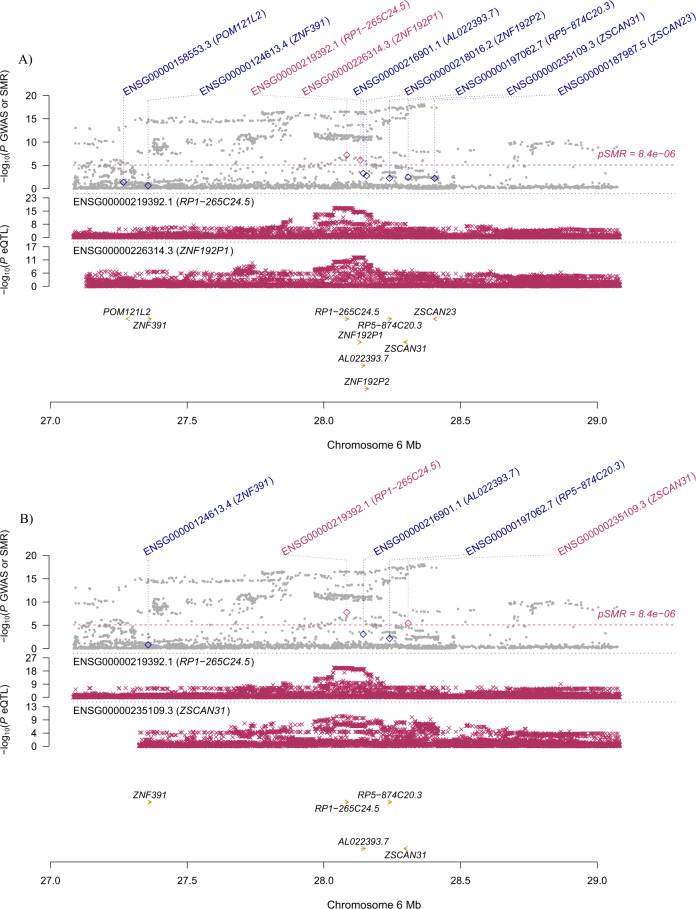
Prioritizing genes around *RP1-265C24.5* in pleiotropic association with MDD. **A** Cortex. **B** Nucleus accumbens. Top plot, gray dots represent the −log_10_(*P* values) for SNPs from the GWAS of MDD, and rhombuses represent the −log_10_(*P* values) for probes from the SMR test with solid rhombuses indicating that the probes pass HEIDI test and hollow rhombuses indicating that the probes do not pass the HEIDI test. Middle plot, eQTL results for the probe ENSG00000219392.1 tagging *RP1-265C24.5*. Bottom plot, location of genes tagged by the probe. Highlighted in maroon indicates probes that pass the SMR threshold. GWAS genome-wide association studies, MDD major depressive disorder, SMR summary data-based Mendelian randomization, HEIDI heterogeneity in dependent instruments, eQTL expression quantitative trait loci.

Specifically, for *BTN3A2*, the most significantly pleiotropic associations with MDD were detected in two brain regions: caudate nucleus and spinal cord (*β* [SE] = 0.043 [0.008], *P* = 7.76 × 10^−^^8^; *β* [SE] = 0.042 [0.008], *P* = 1.72 × 10^−^^7^, respectively; Fig. 1). It also showed a significantly pleiotropic association with MDD in the four brain regions where it was not the top gene (Supplementary Table S1). *RPL31P12* showed the most significantly pleiotropic association with MDD in cerebellar hemisphere and cerebellum (*β* [SE] = −0.037 [0.006], *P* = 7.53 × 10^−^^11^; β [SE] = −0.033 [0.005], *P* = 1.34 × 10^−^^12^, respectively; Fig. 2). *RP1-265C24.5* showed significantly pleiotropic association in cortex and nucleus accumbens (*β* [SE] = 0.036 [0.007], *P* = 6.13 × 10^−^^8^; *β* [SE] = 0.036 [0.006], *P* = 1.63 × 10^−^^8^, respectively; Fig. 3).

The complement gene *C4A* (ENSG00000244731.3) was significantly associated with MDD in 7 different brain regions, after correction for multiple testing (Supplementary Table S1). Of note, both *BTN3A2*, *C4A* and *RP1-265C24.5* are on chromosome 6 while *RPL31P12* is on chromosome 1. Two brain regions, the cerebellar hemisphere and cerebellum, have a relatively large number of significant genes (21 genes and 30 genes, respectively; Table 2).

GO enrichment analysis of biological process and molecular function showed that the significant genes across the different brain regions were involved in four GO terms, including negative regulation of endopeptidase activity (GO:0010951), adaptive immune response (GO:0002250), platelet degranulation (GO:0002576), and negative regulation of defense response (GO:0031348; Supplementary Fig. S2A). Concept network analysis of the identified genes revealed multiple domains related to immune response (Supplementary Fig. S2B). More information could be found in Supplementary Table S2.

Using brain-eMeta eQTL data, we found 75 genes that showed pleiotropic association with MDD, after correction for multiple testing. Specifically, we identified *BTN3A2* (ENSG00000186470) that showed the most significantly pleiotropic association with MDD (*β* [SE] = 0.027 [0.004], *P* = 3.44 × 10^−^^12^; Supplementary Table S3), followed by *RPL31P12* (ENSG00000227207, *β* [SE] = −0.039 [0.006], *P* = 3.43 × 10^−^^11^). We found that *C4A* and *RP1-265C24.5* also showed significant pleiotropic association with MDD (*β* [SE] = 0.031 [0.005], *P* = 1.58 × 10^−^^8^ and *β* [SE] = 0.047 [0.008], *P* = 2.11 × 10^−^^9^, respectively).

GO enrichment analysis of biological process and molecular function showed that the significant genes in brain-eMeta were involved in eight GO terms, including allograft rejection (ko05330), butyrophilin (BTN) family interactions (R-HSA-8851680), platelet degranulation (GO:0002576), immunoregulatory interactions between a lymphoid and a non-lymphoid cell (R-HSA-198933), nuclear chromosome segregation (GO:0098813), telomere maintenance (GO:0000723), organelle localization by membrane tethering (GO:0140056) and lipid transport (GO:0006869; Supplementary Fig. S3A). Similar to the findings for the different brain regions, concept network analysis in brain-eMeta also revealed multiple domains related to immune response (Supplementary Fig. S3B). More information could be found in Supplementary Table S4.

## Discussion

In this study, we integrated GWAS and eQTL data in the MR analysis to explore putative genes that showed pleiotropic/potentially causal association with MDD. Across the different brain regions, we identified multiple significant genes that may be involved in the pathogenesis of MDD. The identified genes were mainly involved in the immune response. Our findings provided important leads to a better understanding of the mechanisms underlying MDD and revealed potential therapeutic targets for the effective treatment of MDD.

Compared to a previous study that adopted a similar SMR approach by integrating GWAS results for MDD and a meta-analyzed version of the eQTL summary data (brain eMeta) to explore causal genes for MDD, our study used the GWAS summary data which were based on a much larger sample size (807,553 vs. 42,455), and we explored the potential pleiotropic association across 13 brain regions^21^. Using brain eMeta, we not only confirmed the significantly pleiotropic association of the three candidate genes (*NEGR1*, *BAG6*, and *HLG-B*) as reported by the study but also identified many other genes (Supplementary Table S3). Moreover, we found many genes showing pleiotropic association with MDD across different brain regions (Table 2 and Supplementary Table S1), representing putative novels genes underlying the pathogenesis of MDD. Our findings suggested that the etiology of MDD involved different genes across different brain regions.

Several of the identified genes in our study, such as *BTN3A2*, *BTN3A3*, *PRSS16*, *HLA-C*, *C4A*, and *HLA-DMA*, are located in or around the human major histocompatibility complex (MHC) region on chromosome 6. MHC represents the most complex genomic region due to its unintelligible linkage disequilibrium^26^. Many genes in or around MHC play an important role in immune response and immune regulation and are involved in a variety of inflammatory and autoimmune diseases^27–31^. The MHC regions can be roughly divided into three classes that are functionally distinct, with class I and II regions containing highly polymorphic human leukocyte antigen (HLA) genes associated with autoimmune disease risk^32,33^ and class III region containing complement component 4 regions associated with schizophrenia risk^34^. Recent GWASs identified a number of genetic variants in the MHC region associated with depression risk, with the strongest association observed in or near the class I region^9–11^.

We found that *BTN3A2* was significantly associated with MDD across many brain regions. *BTN3A2*, which encodes a member of the immunoglobulin superfamily, resides in the juxta-telomeric region (class I) of MHC^35^. The BTN3A2 protein may be involved in adaptive immune response^36^. Previous studies showed that *BTN3A2* was a potential risk gene for Alzheimer’s disease, schizophrenia, and intellectual disability^37–39^. A meta-analysis of GWAS found that *BTN3A2* was associated with neuroticism^40^, an important risk factor for MDD^41^. Overexpression of *BTN3A2* suppressed the excitatory synaptic activity onto CA1 pyramidal neurons, most likely through the interaction with the presynaptic adhesion molecule neurexins^37,42^. Previous research showed that *BTN3A2* was expressed in multiple cell types in the brain, including astrocyte, neuron, oligodendrocyte, and microglia^43^. These findings, together with ours, demonstrated the important role of *BTN3A2* in the nervous system and highlighted the potential of this gene as a promising target for the prevention and treatment of MDD.

A previous GWAS of MDD highlighted the importance of the prefrontal brain regions^10^. In the prefrontal cortex, we found four significant genes, including *BTN3A2*, *C4A*, *RP1-265C24.5*, and *CYP21A1P*, that were associated with MDD after correction for multiple testing. The gene *C4A* was significant in seven brain regions and in the analysis using brain-eMeta. *C4A* localizes to the MHC class III region and encodes the acidic form of complement factor 4. In the mouse brain, *C4A* gene is mainly expressed in astrocytes and neurons^44^. *C4A* is involved in the classical complement activation pathway^45^ and was reported to be associated with schizophrenia, aging, and Alzheimer’s disease^34,46,47^. Moreover, genetic variants in *BTN3A2* and *C4A* were in different LD blocks, suggesting that both genes might be independent risk factors for mental disorders such as schizophrenia and MDD^37^.

Both MDD and schizophrenia are mental illnesses contributing substantially to the global disease burden. It was reported that depressed patients had a higher risk of developing psychosis. Moreover, even prior to the emergence of psychotic symptoms, patients with a high risk of schizophrenia had a higher risk for developing depressive symptoms^48^. In consistent with previous findings^49^, some of the identified genes showing pleiotropic association with MDD were also associated with schizophrenia, such as *BTN3A2*, *BTN3A3*, *PRSS16*, *HLA-C*, *C4A*, and *HLA-DMA*, indicating a potential overlapped mechanism between schizophrenia and MDD.

Our study has some limitations. The number of probes used in our SMR analysis was limited for some brain regions (Table 1), and we may have missed some important genes. The HEIDI test was significant for some of the identified genes, indicating the possibility of horizontal pleiotropy (Supplementary Tables S1 and S3), i.e., the identified association might be due to two distinct genetic variants in high linkage disequilibrium with each other. In addition, we only included study participants of European ethnicity, and our findings might not be generalized to other ethnicities. More studies are needed to validate our findings in independent populations. We adopted correction for multiple testing to reduce the false-positive rate; however, we may have missed important genes. Due to a lack of individual eQTL data, we could not quantify the changes in gene expression in subjects with MDD in comparison with the control.

In conclusion, our SMR analysis revealed that multiple genes showed pleiotropic association with MDD across the brain regions. More studies are needed to explore the underlying physiological mechanisms in the etiology of MDD.

## Supplementary information

Supplementary figure legends

Supplementary Figure 1

Supplementary Figure 2

Supplementary Figure 3

Supplementary tables

## Data Availability

The R and shell scripts used for the analyses are available from the corresponding authors on reasonable request.

## Data availability

All data generated or analyzed during this study are included in this published article and its Supplementary Information files.
