# Supplementary material for: Mendelian randomization integrating GWAS and eQTL data revealed genes pleiotropically associated with major depressive disorder
Source: Transl Psychiatry. 2021 Apr 17;11:225. doi: 10.1038/s41398-021-01348-0 (PMC8053199; doi:10.1038/s41398-021-01348-0)
Supplement: Supplementary file 1 — Supplementary figure legends [file 41398_2021_1348_MOESM1_ESM.docx]

**Figure S1. Distribution of genes showing significantly pleiotropic association with MDD.**

A) A lobe view of the distribution of the significant genes; B) A sagittal view of the distribution of the significant genes

Plots were generated using the R package cerebroViz. Results for cerebellar hemisphere, cortex, nucleus accumbens and spinal cord were not plotted because they were not covered in the package.

AMY, amygdala; CAU, caudate; CB, cerebellum; CNG, anterior cingulate cortex; FL, frontal cortex; HIP, hippocampus; HTH, hypothalamus; MDD, major depressive disorder; PUT, putamen; SN, substantia nigra.

**Figure S2. Functional enrichment and gene concept network analysis based on the identified genes in different brain regions.**

A) Enriched GO terms based on the identified genes in different brain regions; B) Concept network analysis of the identified genes

GO, gene ontology

**Figure S3. Functional enrichment and gene concept network analysis based on the identified genes using brain-eMeta eQTL data.**

A) Enriched GO terms based on the identified genes in brain-eMeta; B) Concept network analysis of the identified genes

GO, gene ontology; eQTL, expression quantitative trait loci
