# Supplementary figures and images for: Mendelian randomization integrating GWAS and eQTL data revealed genes pleiotropically associated with major depressive disorder
Source: Transl Psychiatry. 2021 Apr 17;11:225. doi: 10.1038/s41398-021-01348-0 (PMC8053199; doi:10.1038/s41398-021-01348-0)

A

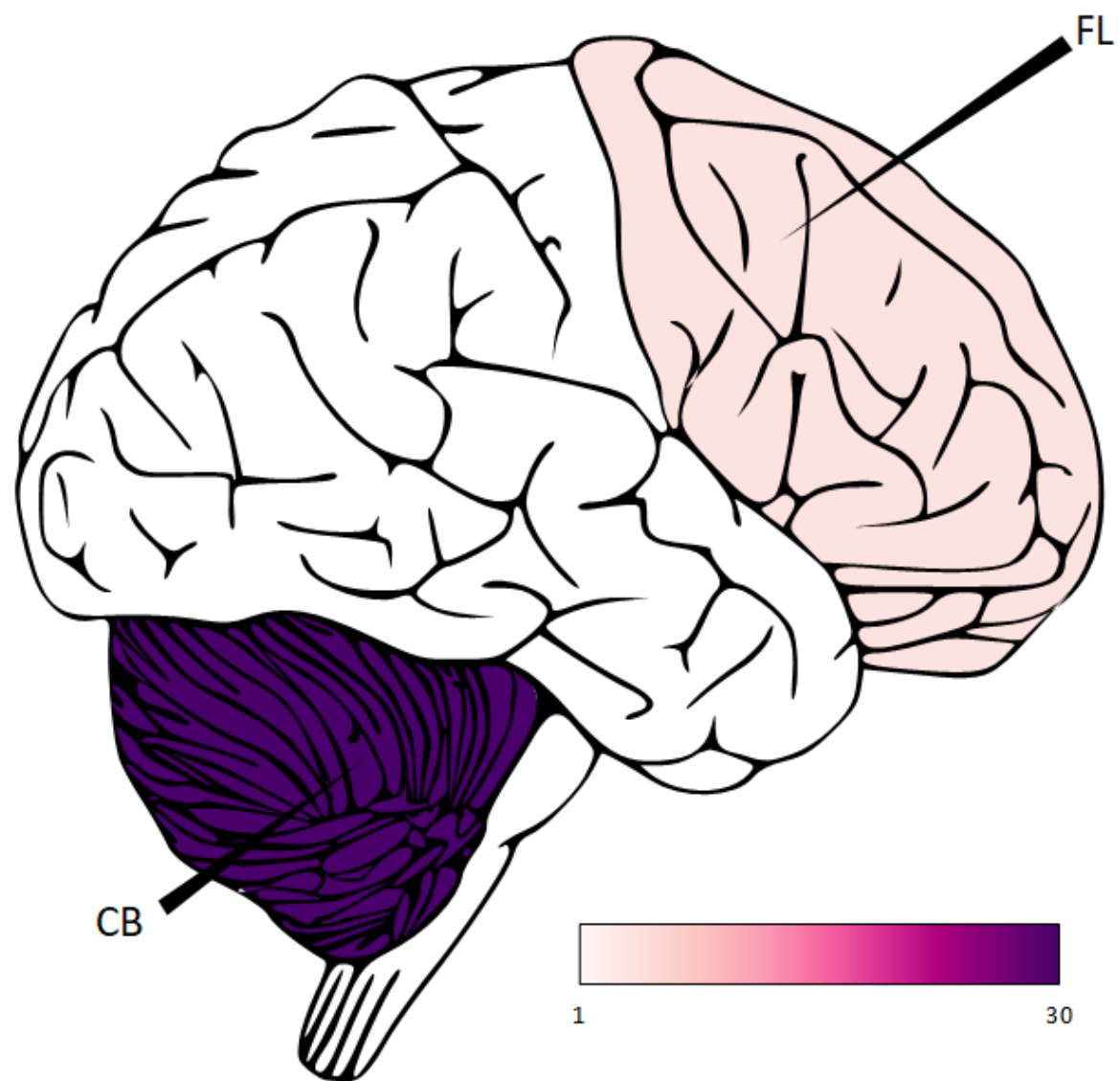

B

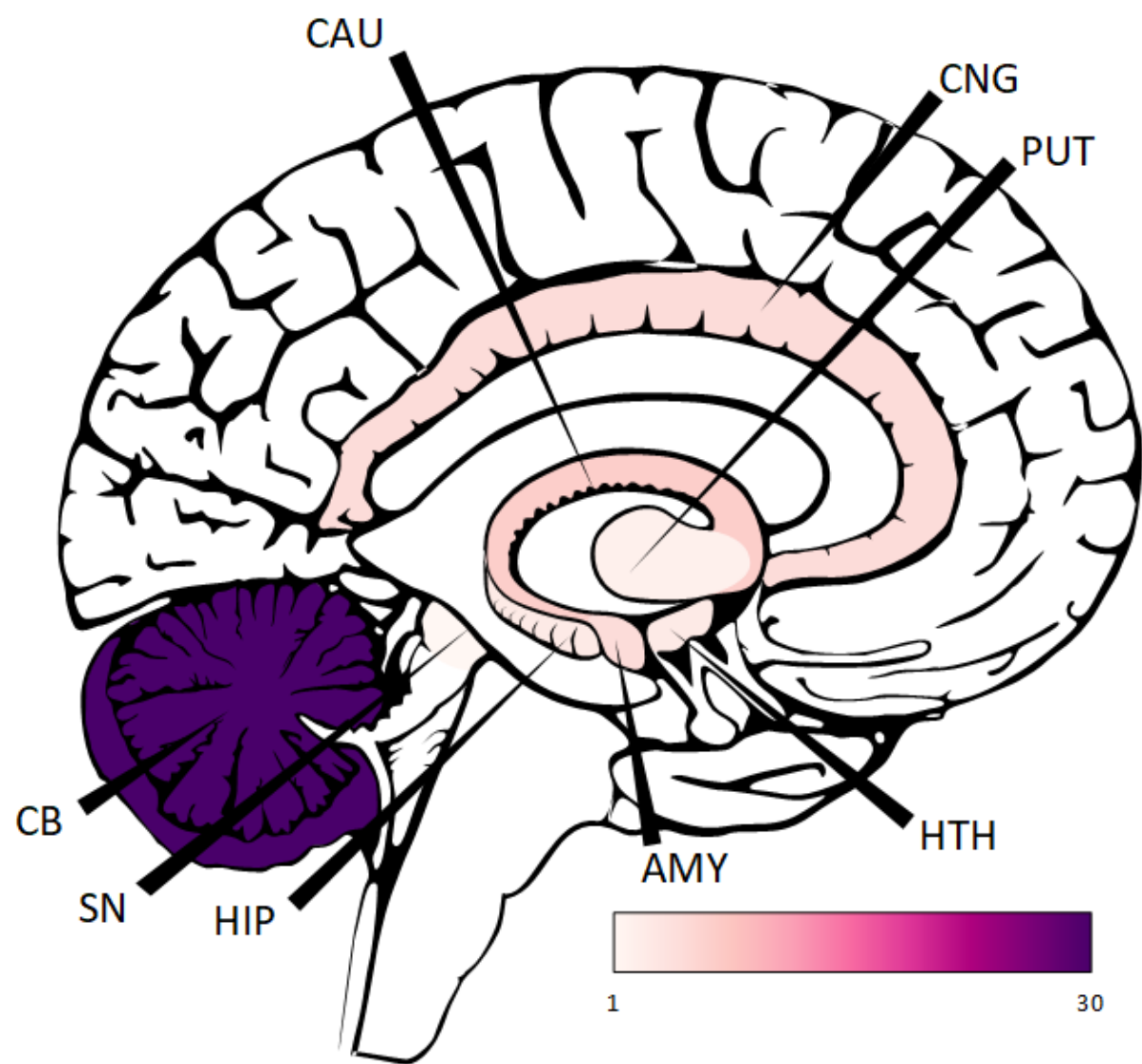

Supplement: Supplementary file 2 — Supplementary Figure 1 [file 41398_2021_1348_MOESM2_ESM.pdf]

**A**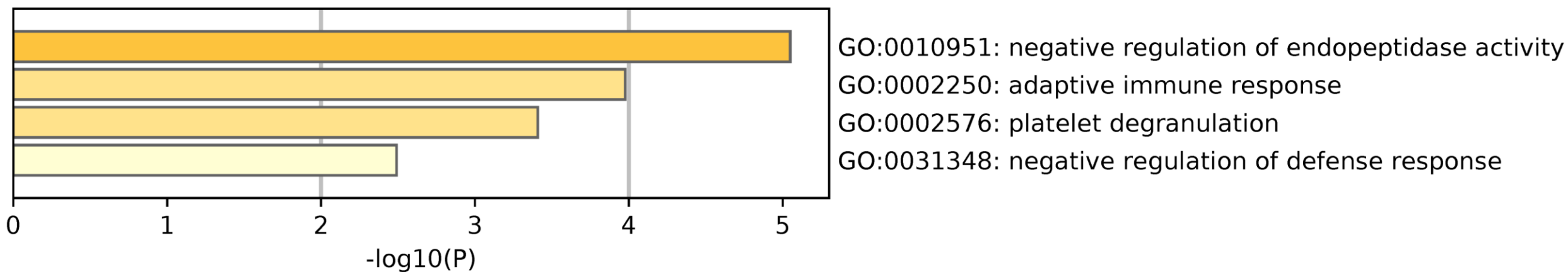**B**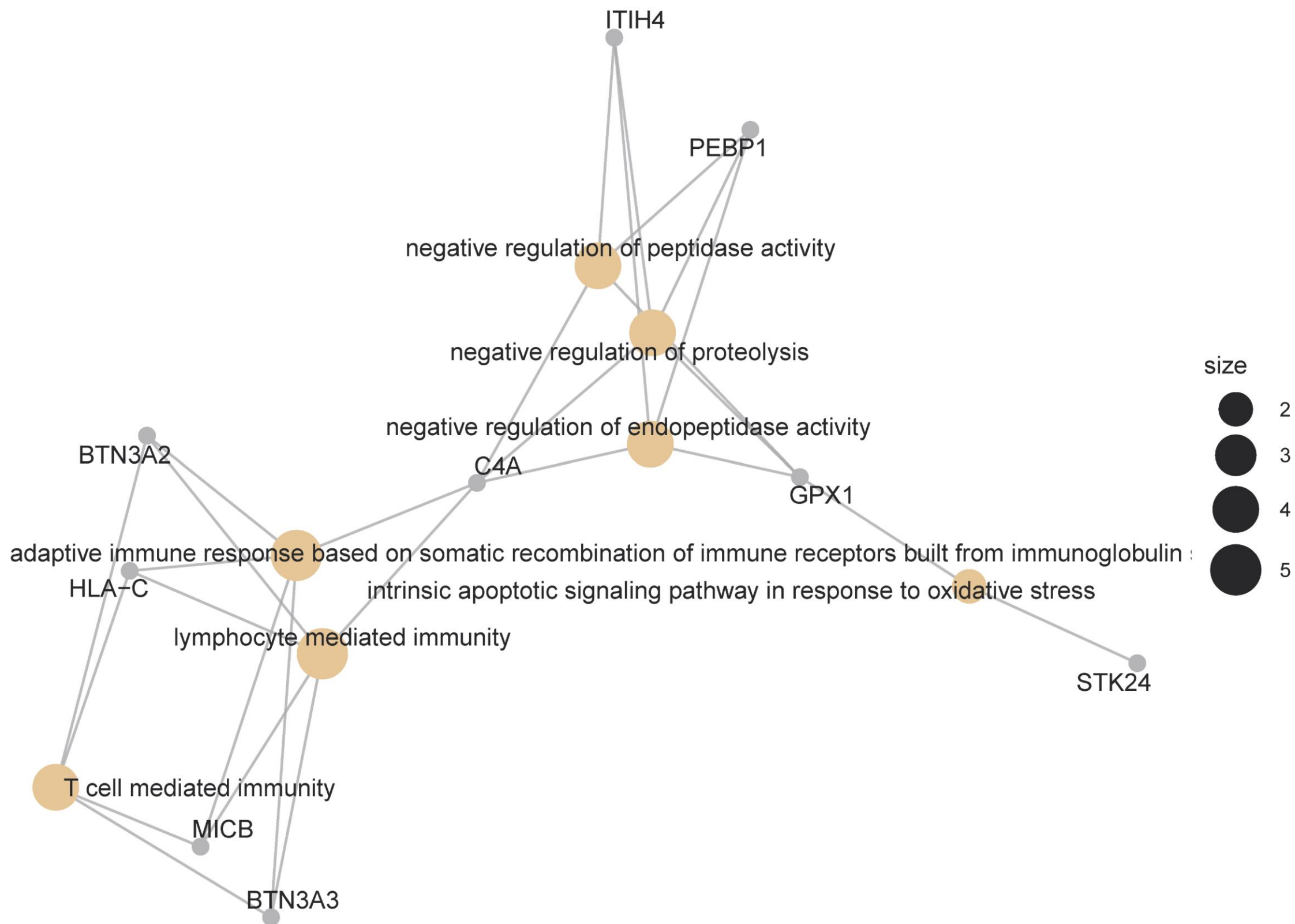

Supplement: Supplementary file 3 — Supplementary Figure 2 [file 41398_2021_1348_MOESM3_ESM.pdf]

**A**

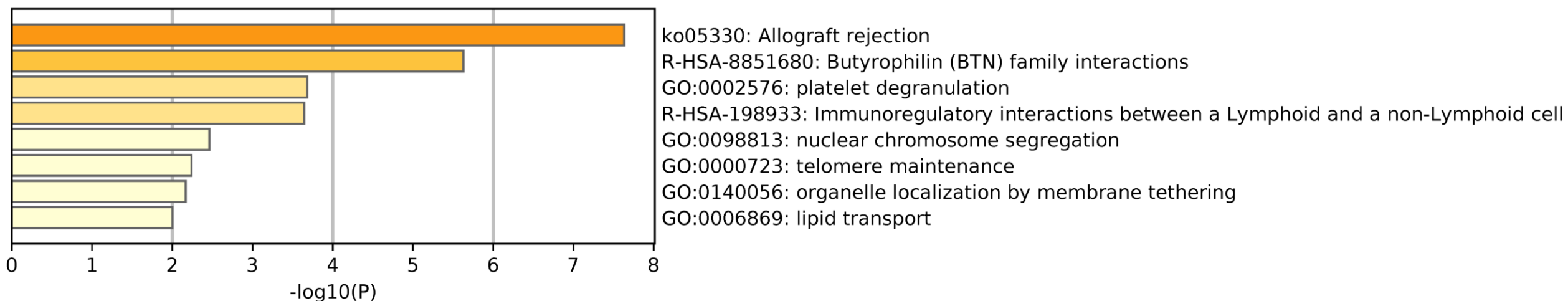

**B**

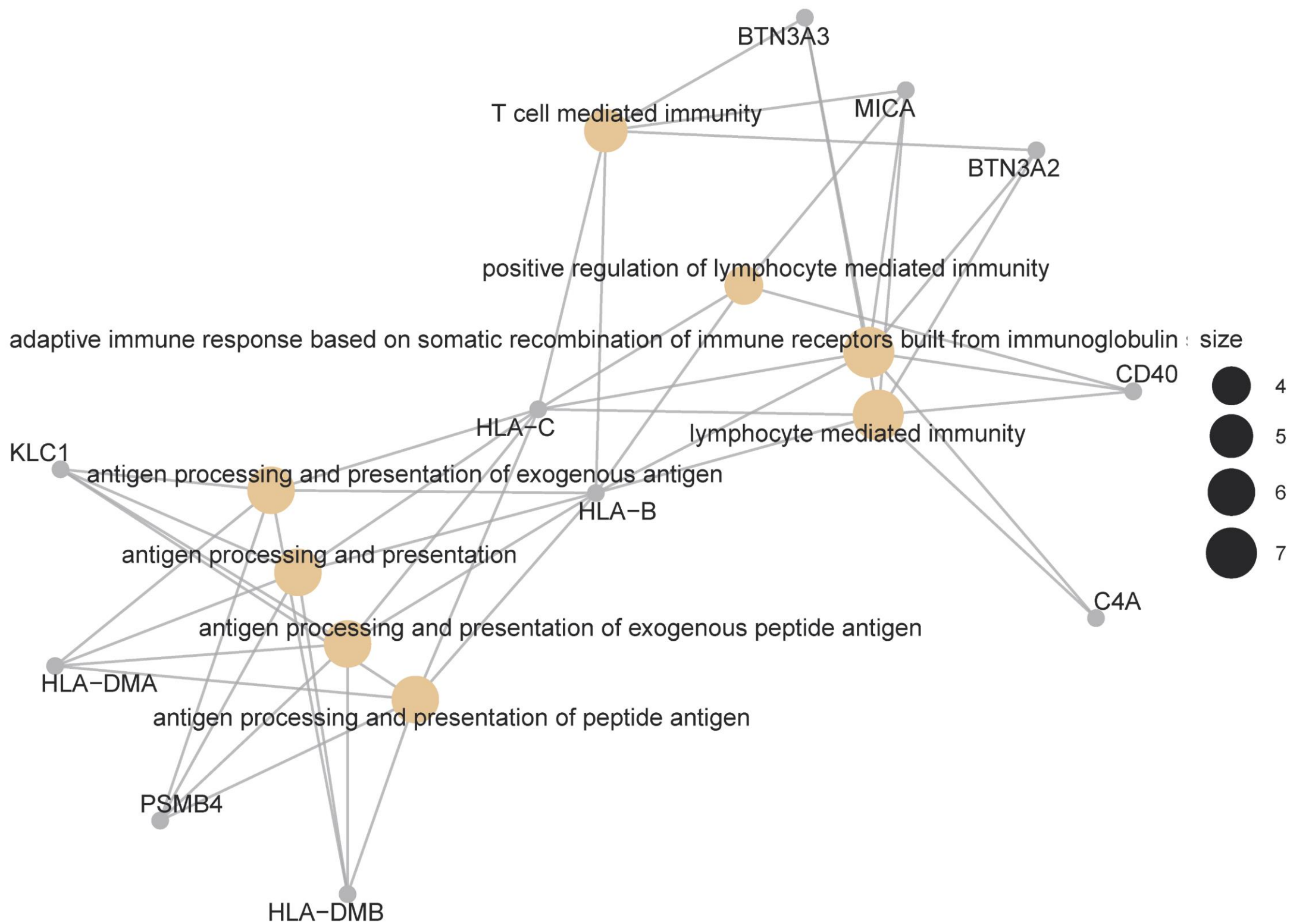

Supplement: Supplementary file 4 — Supplementary Figure 3 [file 41398_2021_1348_MOESM4_ESM.pdf]
